# Neuroprotective Effect of Quercetin Nanocrystal in a 6-Hydroxydopamine Model of Parkinson Disease: Biochemical and Behavioral Evidence

**DOI:** 10.32598/bcn.9.5.317

**Published:** 2018-09-01

**Authors:** Fatemeh Ghaffari, Akbar Hajizadeh Moghaddam, Mahboobeh Zare

**Affiliations:** 1. Department of Biology, Faculty of Basic Sciences, University of Mazandaran, Babolsar, Iran.; 2. Department of Medicinal Plants, School of Science and Herbs, Amol University of Special Modern Technologies, Amol, Iran.

**Keywords:** Parkinson disease, Antioxidant, Quercetin, Nanocrystal, 6-Hydroxydopamine

## Abstract

**Introduction:**

Studies have suggested that free radicals-induced neurodegeneration is one of the many studies of Parkinson Disease (PD). Quercetin as a natural polyphenol has been regarded as a significant player in altering the progression of neurodegenerative diseases by protecting from damages caused by free radicals. Owing to its poor water solubility, preparation of its oral formulation is urgently needed. Recently, nanocrystal technique as an effective way has been introduced for oral administration of drugs.

**Methods:**

This study investigated the neuroprotective effects of quercetin nanocrystals on 6-hydroxydopamine (6-OHDA)-induced Parkinson-like model in male rats. Quercetin nanocrystals were prepared by the Evaporative Precipitation of Nanosuspension (EPN) method.

**Results:**

Administration of quercetin and its nanocrystals (10 and 25 mg/kg) prevented disruption of memory, increased antioxidant enzyme activities (superoxide dismutase and catalase) and total glutathione and reduced Malondialdehyde (MDA) level in the hippocampal area.

**Conclusion:**

The present study results demonstrated that quercetin nanocrystals with greater bioavailability is effective than quercetin alone in treatment of Parkinson-like model in rat.

## Highlights

- Hydroxydopamine (6-OHDA) injection induces behavioral impairment and oxidative stress in hippocampal area.
- Quercetin and its nanocrystal treatment improves 6-OHDA-induced memory impairment and hippocampal oxidative stress.
- In the behavioral and biochemical experiments, nano-quercetin treatment shows additional distinguished results compared to quercetin and it could be a beneficial neuro-protection applicant for Parkinson treatment.

## Plain Language Summary

Recent studies indicate that oxidative stress is a critical factor in Parkinson Disease. Oxidative stress caused by free radical overproduction can damage the brain. Quercetin, a natural flavonoid molecule, is present in vegetables and fruits such as tea, apples, mulberries, onions, broccoli, peanuts and red wine. Because of the low absorption of quercetin in small intestine, the therapeutic use of this compound is limited. Thus, the present study aimed to analyze the neuro-protective role of quercetin nano-crystals and compare of its effect with coarse quercetin in a rat model of Parkinsonism. The result shown that 6-hydroxydopamine induced recognition memory deficits and oxidative stress in rats brain, while treatment with quercetin and its nano-crystals were improved recognition memory deficits and increased hippocampal antioxidant parameters by scavenging free radicals.

## 1. Introduction

Parkinson Disease (PD), as the most common age-related neurodegenerative disease, is characterized by the loss of dopaminergic neurons in the Substantia Nigra pars compacta (SNpc), with clinical symptoms, including bradykinesia, tremor, and rigidity (Shim et al., 2009). Recent studies in PD brains indicate that oxidative stress is a critical factor in pathogenesis of PD (Hritcu & Ciobica, 2013; Miller et al., 2009; Danielson & Andersen, 2008). Oxidative stress results from an imbalance between Reactive Oxygen Species (ROS) production and activity of antioxidant systems, which can cause damage to proteins, lipids, and DNA (Dong et al., 2014).

6-Hydroxydopamine (6-OHDA) is used to produce animal models of PD. This neurotoxic compound induces apoptotic activity through the formation of various reactive oxygen species, lipid peroxidation, damaged protein, and amino acid modification (Kuruvilla, Nandhu, Paul, & Paulose, 2013; Hritcu, Ciobica, & Artenie, 2008; Hritcu et al., 2011; Ciobica, Hritcu, Artenie, Stoica, & Bild, 2009). Antioxidants present in food may protect the cell components against oxidative damage and therefore limit the risk of various degenerative diseases associated with the oxidative stress (Scalbert, Manach, Morand, Rémésy, & Jiménez, 2008). Flavonoids are the most abundant antioxidants in the diet and are widespread in medicinal plants, vegetables, and fruit juices (Bakkialakshmi & Barani, 2013).

Quercetin, a natural flavonoid molecule, is present in vegetables and fruits such as tea, apples, mulberries, onions, broccoli, peanuts, and red wine (Dong et al., 2014). This polyphenolic flavonoid has attracted the researchers’ interest because of its wide availability and potent biologically active agent to prevent potential problems such as cardiovascular diseases (Kamada, da Silva, Ohnishi-Kameyama, Moon, & Terao, 2005), anti-inflammatory (Cho et al., 2003), and neurodegenerative disorders (Dhawan, Kapil, & Singh, 2011). In addition, it is postulated to act as a novel neuroprotective by radical scavenging of reactive oxygen species (Shanmugam, Gowthamarajan, Priyanka, Madhuri, & Karri, 2013). Although quercetin has many therapeutic effects, its application in the pharmaceutical field is limited because of low solubility and bioavailability (Kakran et al., 2012a). To overcome these problems, nanoquercetin is applied to maximize the efficiency in the treatment of various diseases such as PD (Ganesan, Ko, Kim, & Choi, 2015; Díaz, Vaamonde, & Dajas, 2015).

Nanosizing of phytomedicines enhances the permeability into the brain with maximized efficiency and stability (Ganesan, et al., 2015; Díaz, et al., 2015). Over the last few years, nanocrystallization has been successfully applied to deliver poorly soluble drugs. Nanocrystals can increase the saturation solubility and the dissolution rate of drug particles due to its small size and the high surface area to volume ratio (Sun & Yeo, 2012; Kakran et al., 2012b). There are several methods to produce drug nanoparticles such as jet mill, spray freezing, Evaporative Precipitation of Nanosuspension (EPN) and high pressure homogenization.

EPN is a new method that relies on high super saturation and homogeneous nucleation to obtain drug nanocrystals. The simplicity and low setup cost are advantages of this method (Shanmugam et al., 2013). Therefore, the current study aimed at analyzing the neuroprotective role of quercetin nanocrystal and comparing its effect with that of coarse quercetin in a rat model of PD.

## 2. Methods

### 2.1. Animals

The current study was conducted on 49 male rats (weighing 230±10 g) kept in animal room with a 12:12 hour light/dark cycle at 22±2°C. They had free access to food and tap water except during the time of experiments. All animals were allowed to adapt to the laboratory conditions for at least one week before surgery.

### 2.2. Chemical compounds

Quercetin, 6-OHDA, apomorphine, glacial acetic acid and reduced Glutathione (GSH) were purchased from Sigma-Aldrich Chemical Company (USA), and potassium dihydrogen phosphate, sodium dihydrogen phosphate (NaH-2PO4), Trichloroacetic Acid (TCA), Thiobarbituric Acid (TBA), and hydrogen peroxide from MERK Company (Germany). The other chemicals used were of grade-purity.

### 2.3. Preparation of quercetin nanocrystal

Quercetin nanocrystal was prepared by the reported procedure (Kakran et al., 2011). Briefly, quercetin was dissolved in ethanol (5 mg/mL) and hexane was quickly added to it (ethanol to hexane ratio was 1:25 by volume). Quercetin nanocrystal was obtained by evaporation of the solvents by rotary evaporator. The particle size and morphology of samples was observed using a scanning electron microscope (JSM–5510-SEM, Jeol Co. Japan).

### 2.4. Animal treatment

In the current study, male adult rats were randomly divided into seven groups; the control group, the vehicle group (gavaged by distilled water and received intrastriatal injection of sterile normal saline), the lesioned group (gavaged by distilled water and received intrastriatal injection of 10 μg 6-OHDA/2μL in 0.1% ascorbic acid-saline), two quercetin-treatment lesioned groups (10 and 25 mg/kg body weight), and two quercetin nanocrystal-treatment lesioned groups (10 and 25 mg/kg body weight). The drug dissolved in the normal saline was administered by gavage daily, for four weeks after neurosurgery.

### 2.5. Stereotaxic surgery

Rats were anaesthetized intraperitoneally with ketamine hydrochloride (50 mg/kg) and xylazine (4 mg/kg) and placed in a Stoelting stereotaxic instrument (Stoelting Co, Illinois, USA). According to Paxinos and Watson (1998), stereotaxic coordinates for injection into the striatum were +1 mm anterior to bregma, 2.5 mm lateral to the midline, and 4.5 mm ventral of the dorsal surface of the skull. All the animals in the lesioned group and treatment lesioned groups received a 10 μg 6-OHDA 2 μL in 0.1% ascorbic acid-saline injection into the right striatum (Debeir et al., 2005) for rat, whereas the vehicle group received 2.0 μL of the 0.1% ascorbic acid-saline.

### 2.6. Rotational behavior test

All rats were tested for rotational behavior by apomorphine subcutaneously (0.5 mg/kg in normal saline containing 0.01% ascorbic acid) three weeks after the lesion. The rotations were measured by the Rizelio et al. (2010) method.

### 2.7. Novel object recognition test

Three weeks after 6-OHDA injection, rats were tested for Novel Object Recognition (NOR) test. NOR test was performed as described previously (de Lima et al., 2011). The test apparatus consisted of a dark open box. It was illuminated by a 60 W lamp. Before the test, rats were acclaimed to the apparatus. At the training session, rat was placed in a box with two identical objects for five minutes. After the training session, one similar object was replaced by a new object and rat was returned to the open field for a five-minute test session. Recognition ratio for each rat was expressed by TN/(TN+TF) ratio (TF: Time spent exploring Familiar object, TN: Time spent exploring the Novel object). The percentage of time spent exploring the new object served as the measure of recognition memory for the familiar object.

### 2.8. Biochemical parameter assay

Four weeks after the administration of drugs in 6-OHDA-lesioned groups, all rats were anesthetized, rapidly decapitated, and their whole brains were removed, and the hippocampus areas were collected. Tissue samples were kept at −60°C before biochemical estimations; and 150 g of the hippocampus tissue was homogenized in tris buffer with pH=7.4 and centrifuged at 13600 g for half an hour. The supernatant was used to analyze the hippocampal antioxidant enzyme activities, including Superoxide Dismutase (SOD) and Catalase (CAT), and nano-enzymatic antioxidant indexes, including Glutathione (GSH) and Malondialdehyde (MDA) levels.

### 2.9. Determination of superoxide dismutase activity

SOD assay was performed according to the method introduced by Genet et al. (2002) with some modification. Briefly, 50 mM sodium phosphate buffer was mixed with EDTA (0.1 mM), pyrogallol (0.48 mM) and 20 μL enzymatic extract. Distilled water was added to a final volume of 1 mL. The decrease in absorbance was then followed at 420 nm for one minute at 25°C against a blank containing all the ingredients without the homogenated tissue. One unit of enzyme was defined as the amount of enzyme that causes half maximal inhibition of pyrogallol autoxidation.

### 2.10. Determination of catalase activity

Catalase activity in hippocampus was assayed according to the method used by Genet et al. (2002) at 240 nm for two minutes. Enzymatic reaction was initiated by adding 20 μL enzymatic extract and the substrate of 10 mM hydrogen peroxide in a medium containing 50 mM sodium phosphate buffer (pH 7.0). CAT activity was defined as units (U)/mg protein.

### 2.11. Total glutathione content

The levels of hippocampal Glutathione (GSH) was determined by the method used by Fukuzawa and Tokumura (1976) using (2-nitrobenzoic acid) (DTNB) for color development. Absorbance was recorded at 412 nm and results were expressed as μg/mg protein tissue.

### 2.12. Determination of malondialdehyde

Lipid peroxidation was measured by the method of Herman and Kevin (1989). In brief, the mixture of tissue homogenates including 1 mg protein, TCA (1 mL, 20%), and thiobarbituric acid (2 mL, 0.67%) was incubated for one hour at 100°C. After cooling at room temperature, the precipitate was removed by centrifugation and the absorbance of the supernatant was then measured at 532 nm.

### 2.13. Statistical analysis

One-way ANOVA was performed to compare the effect of various doses of drugs and 6-OHDA exposure on hippocampus area. Following a significant F-value, post hoc analysis (Tukey’s test) was used to compare the difference between groups. The difference was considered statistically when P<0.05. Data were presented as Means±SD.

## 3. Results

### 3.1. Particle size determination

**Figure 1.**
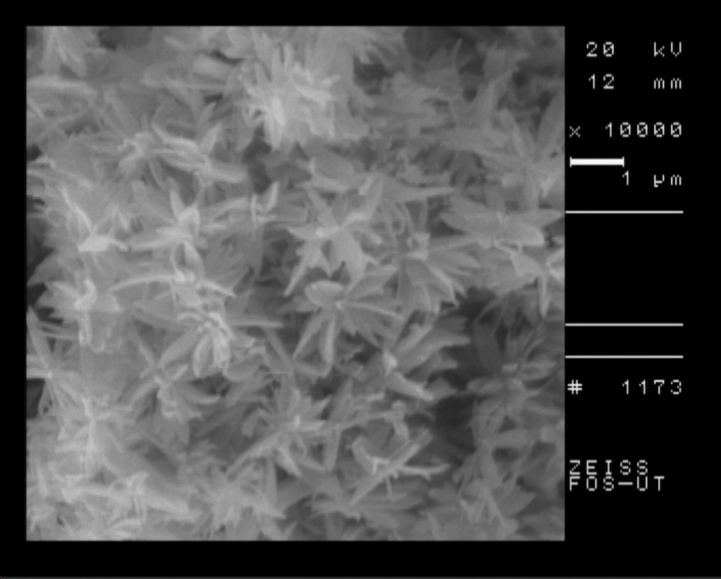
Scanning electron microscope photo of quercetin nanocrystal

The scanning electron micrograph of the quercetin nanocrystal is shown in Figure 1. The morphology of particles was irregular and flake type and the average particle size of quercetin nanocrystal was 120 nm.

### 3.2. Effect of quercetin and quercetin nanocrystal treatment on apomorphine-induced circling behavior in rats

**Figure 2.**
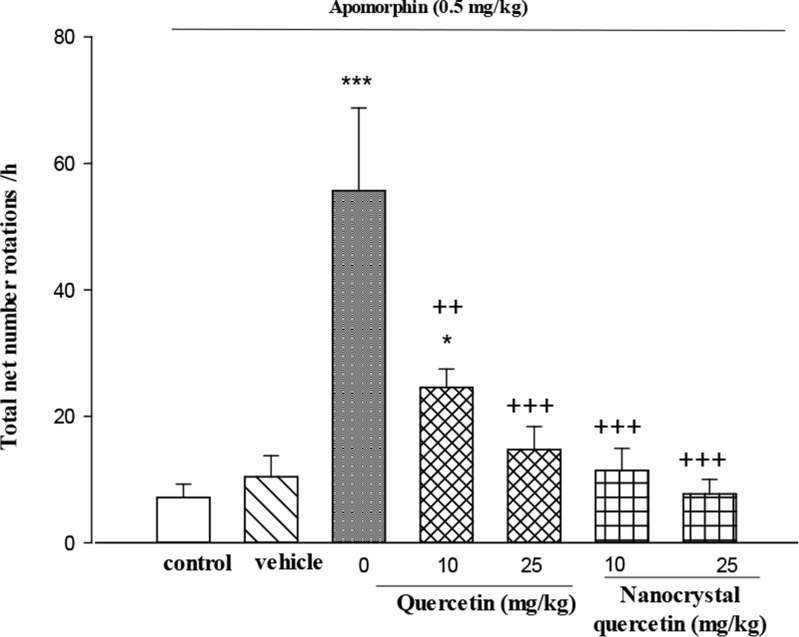
Total net number of rotations induced by apomorphine The data are expressed as Mean±SE (n=7); ^*^ P<0.05 and ^***^ P<0.001 versus control group; ^++^ P<0.01 and ^+++^ P<0.001 versus lesioned group.

The 6-OHDA-induced lesioned rats showed a significant increase (P<0.001) in rotations compared with the control group; 10 mg/kg of quercetin (P<0.01), 25 mg/kg of quercetin (P<0.001) and quercetin nanocrystals (P<0.001) treatment lesioned groups showed a significant decrease in the rotations compared with the lesioned group (Figure 2).

### 3.3. Effects of quercetin and quercetin nanocrystal treatment in novel object recognition test

**Figure 3.**
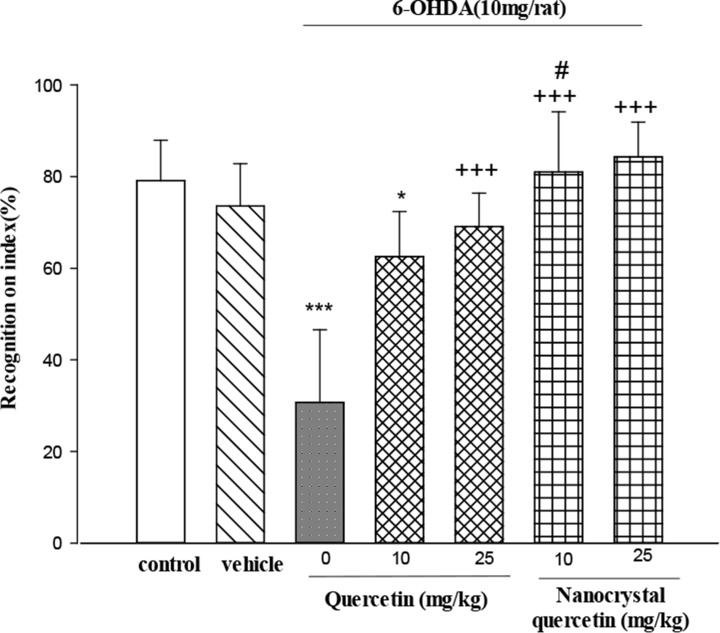
Effects of quercetin and quercetin nanocrystal treatment on rats’ memory performance in the NOR test The data are expressed as Mean±SE (n=7); ^*^ P<0.01 and ^***^ P<0.001 versus control group; ^++^ P<0.01 and ^+++^ P<0.001 versus lesioned group; ^#^ P<0.05 versus 10 mg/kg quercetin group.

The influence of treatment with quercetin nanocrystal on 6-OHDA-induced memory impairment was investigated in the NOR test (Figure 3). As a normal behavior, rats spent less time on the familiar object in the test session compared with training. Histograms in Figure 3 showed that 6-OHDA decreased trial unique recognition memory. The analysis of index R data revealed a significant effect of 6-OHDA (P<0.001). In addition, rats treated with 10 mg/kg quercetin in the treatment lesioned group had a significantly (P<0.01) increased index R compared with those of the lesioned group. In addition, rats treated with 25 mg/kg of quercetin in the lesioned group and quercetin nanocrystals treatment in the lesioned groups had a significantly (P<0.001) increased index R compared with those of the lesioned group.

### 3.4. Effect of quercetin and its nanocrystal on superoxide dismutase and catalase activities

Biochemical analyses showed the increase of the enzymatic antioxidant activities in the hippocampal tissue homogenates of the 6-OHDA-lesioned groups treated with the quercetin and quercetin nanocrystal (Table 1). Results in Table 1 showed a significant increase of the SOD enzyme activity in 6-OHDA-lesioned groups treated with quercetin nanocrystal compared to lesioned group (P<0.05).

**Table 1.** Effect of quercetin and its nanocrystal on hippocampal antioxidant parameters

**Groups**	**SOD (% Inhibition)**	**CAT (U/mg Protein)**	**GSH (μg GSH/μg Protein)**	**MDA (μmol/mg Protein)**
Control	82.87±.2.63	42.22±6.53	0.75±0.04	0.17±0.00
Vehicle	78.54±2.17	36.78±5.55	0.60±0.06	0.16±0.01
6-OHDA	69.84±8.91^***^	3.33±0.56^***^	0.22±0.03^***^	0.24±0.01^***^
Quercetin 10+6-OHDA	63.30±1.32^**^	2.26±0.47^***^	0.28±0.07^***^	0.16±0.01^+++^
Quercetin 25+6-OHDA	61.95±1.01^*^	5.86±1.32^***^	0.25±0.06^***^	0.13±0.00^***+++^
Quercetin nanocrystal 10+6-OHDA	55.37±1.03^*+^	14.25±6.42^***^	0.38±0.03^***^	0.09±0.00^***+++###^
Quercetin nanocrystal 25+6-OHDA	49.30±4.19^+++^	26.33±3.97^++#^	0.51±0.03^**++#^	0.09±0.00^***+++^

The data are expressed as Mean±SE (n=7).

* P<0.05,

** P<0.01, and

*** P<0.001 versus control group.

+ P<0.05,

++ P<0.01, and

+++ P<0.001 versus lesioned group.

# P<0.05 versus quercetin (25 mg/kg) group;

### P<0.001 versus quercetin (10 mg/kg) group.

A significant increase of the CAT enzyme activity (P<0.01) (Table 1) was also observed in 6-OHDA-lesioned groups treated with 25 mg/kg of the quercetin nanocrystal compared with the lesioned group. In addition, there were no significant differences between the control, vehicle, and quercetin nanocrystal (25 mg/kg) treatment groups. In addition, post hoc analysis revealed significant differences between quercetin (25 mg/kg) and quercetin nanocrystal (25 mg/kg) treatment-lesioned groups (P<0.05).

### 3.5. Effect of quercetin and its nanocrystal on total glutathione content and malondialdehyde level

According to the results in Table 1, 6-OHDA decreased glutathione levels in hippocampal tissue homogenates as compared with those of the control group (P<0.001). The treatment of lesioned groups with quercetin nanocrystal at high dose (25 mg/kg) showed significant increase in GSH level as compared to lesioned group (P<0.01) and the same dose of quercetin treatment (P<0.05) groups. In addition, post hoc analysis revealed significant differences between high dose of quercetin and quercetin nanocrystal treatment lesioned groups (P<0.05).

Table 1 showed that 6-OHDA increased lipid peroxidation level of hippocampal tissue homogenates compared with those of the control group (P<0.001). Quercetin and quercetin nanocrystal treatment showed significant decrease in MDA level as compared with those of the lesioned group (P<0.001). Also quercetin (25 mg/kg) and quercetin nanocrystal (10 and 25 mg/kg) treatment showed significant decrease in MDA level as compared with those of the control group (P<0.001). On the other hand, the treatment of lesioned groups with 10 mg/kg quercetin nanocrystal showed significant decrease in MDA level as compared with those of the same dose of quercetin treatment group (P<0.001).

## 4. Discussion

The damage of free radicals to neuron components such as DNA, proteins, and lipids increases with age and contributes to the degeneration of the somatic neuron, and leads to neurodegenerative diseases (Shim et al., 2009). Flavonoids such as quercetin can help limit the damage due to their antioxidant properties or by stimulating internal defense systems (Scalbert et al., 2005). Quercetin has low bioavailability because of its poor solubility and membrane permeability. Therefore, the clinical application of this drug is restricted. Fabricating quercetin nano-crystal can greatly increase its saturation solubility and the dissolution velocity.

The nanocrystal technology attracted the attention of a lot pharmaceutical scientists in the drug delivery system due to the increase of the surface area, and thus the dissolution velocity. The ability to produce molecules as nano size particles can have a significant effects on efficiency such as increasing bioavailability, allowing for dosage decrease, and improving efficacy (Junghanns & Müller, 2008). In the current study, the quercetin nanocrystal was prepared by EPN and the neuroprotective effect of quercetin and its nanocrystals were investigated in 6-OHDA- lesioned rat models of PD.

In the rotational behavior test, quercetin and its nanocrystals reduced the apomorphine-induced rotation. Several studies reported that injection of 6-OHDA into the striatum enhanced contralateral apomorphine-induced rotation by a reduction in the striatal dopamine level and an up-regulation of dopaminergic postsynaptic receptors at the same side (Choi et al., 2010). Thus, decrease in rotation may be due to a protective effect of these drugs on dopaminergic neurons against 6-OHDA toxicity. Traditionally, PD is considered a motor disorder but recently the nature of cognitive impairment, ranging from minor disturbances in memory to various intellectual functions and dementia are of interest in PD (Lefter et al., 2014).

The novel object recognition task is widely used to assess recognition memory performance in rodents (de Lima et al., 2011). It is employed to test the effects of various pharmacological treatments and brain damage (Antunes & Biala, 2012). Therefore, the current study investigated the effects of quercetin and quercetin nanocrystal treatment on memory deficits by novel object recognition task and the obtained results showed that 6-hydroxydopamine induced recognition memory deficits in rats, while treatment with original quercetin and its nanocrystal were effective on preventing the impairment of memory.

6-Hydroxydopamine can produce intracellular H2O2. This leads to the production of reactive hydroxyl radicals and decreased activities of antioxidant enzymes such as SOD and CAT, total GSH content, and increased lipid peroxidation (MDA level) (Hritcu et al., 2008; Hritcu et al., 2011; Ciobica et al., 2009) as observed in the current study. Additionally, in the current study, the quercetin and its nanocrystals restored the activity of SOD and CAT, GSH level, and decreased lipid peroxidation in the hippocampal area. This result demonstrated that increased antioxidant enzyme activities can lead to decreased production of intracellular H2O2, with a simultaneous increase of GSH level, this could decrease the lipid peroxidation; implying that quercetin nanocrystal possesses strong antioxidant property.

According to these results, lesioned rats treated with quercetin nanocrystal showed better results in memory recognition and regulation of lipid peroxidation, maintenance of glutathione levels, and antioxidant enzyme activities in the brain homogenates compared to the rats treated by equal doses of coarse quercetin. The previous studies demonstrated that the quercetin nanocrystals showed higher free radical scavenging toward the coarse quercetin (Sun & Yeo, 2012; Kakran et al., 2012b; Krishnakumar et al., 2015). The current study observed that the neuroprotective effect of quercetin improved in its nanocrystal form due to increase in particle bioavailability.

In summary, quercetin and its nanocrystals displayed neuroprotective effect against the damage following 6-OHDA in experimental rats. Neurobehavioral deficits improved and oxidative stress induced in hippocampal area was significantly protected following quercetin nanocrystal administration. The mechanism could be partly attributed to antioxidant scavenging free radicals. Quercetin nanocrystal indicated higher bioavailability in-vivo compared with coarse quercetin, which might lead to a dose reduction for patients.

## Ethical Considerations

### Compliance with ethical guidelines

All procedures of the current study were performed according to the Research Ethics Committee of University of Mazandaran.
